# 3-Nitro­benzo­nitrile

**DOI:** 10.1107/S2414314623008143

**Published:** 2023-09-22

**Authors:** Miha Virant, Ana Siljanovska, Janez Cerkovnik, Matic Lozinšek

**Affiliations:** a Jožef Stefan Institute, Jamova cesta 39, 1000 Ljubljana, Slovenia; b Faculty of Chemistry and Chemical Technology, University of Ljubljana, Večna pot 113, 1000 Ljubljana, Slovenia; University of Aberdeen, United Kingdom

**Keywords:** nitro, nitrile, benzo­nitrile, crystal structure

## Abstract

The crystal structure of 3-nitrobenzonitrile, C_7_H_4_N_2_O_2_, was elucidated by low-temperature single-crystal X-ray diffraction. The compound crystallizes in the Sohncke space group *P*2_1_ and features two mol­ecules in the unit cell. Aromatic π–π stacking leads to stacks of mol­ecules in the [100] direction. The absolute structure was established from anomalous dispersion.

## Abstract 2

3-Nitro­benzo­nitrile crystallizes in the Sohncke space group *P*2_1_.

## Graphical abstract

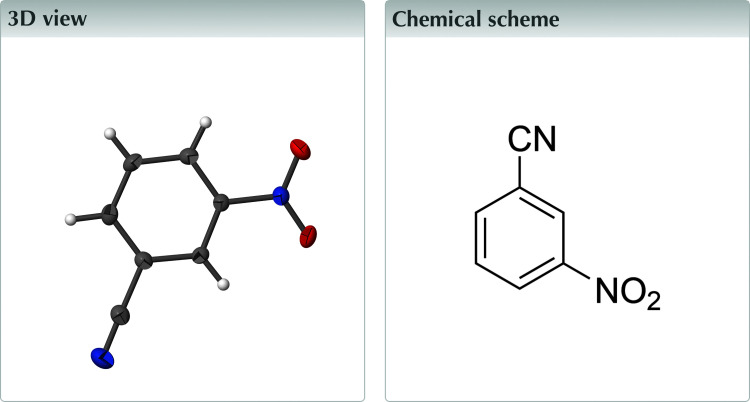

## Structure description

**Figure 1:**
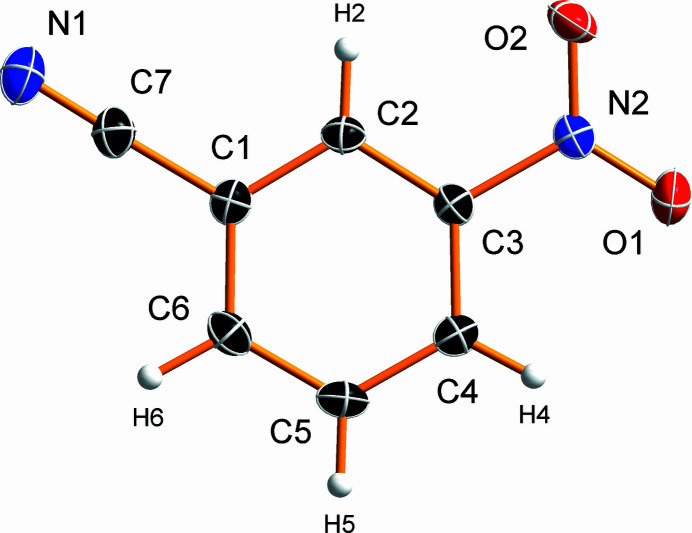
The mol­ecular structure of the title compound showing 50% displacement ellipsoids. Hydrogen atoms are depicted as spheres of arbitrary radius.

3-Nitro­benzo­nitrile crystallizes in the monoclinic Sohncke space group *P*2_1_ with one mol­ecule in the asymmetric unit (Fig. 1 ▸). The nitro group is not coplanar with the benzene ring, but slightly tilted. The corresponding angle between the benzene ring and NO_2_ plane normals is 11.22 (6)° with atom O1 located 0.163 (3) Å below and atom O2 0.253 (3) Å above the plane of the benzene ring.

This tilt of the NO_2_ group is presumably the result of the crystal packing, which locks the orientation of the NO_2_ group. The corresponding non-coplanar orientation of the NO_2_ group induces the asymmetry of the mol­ecule and, in turn, the chirality of the crystal. In solution, where the barriers for the rotation of the nitro group are usually low, for instance, 19 kJ mol^−1^ in nitrobenzene determined by gas-phase electron diffraction (Borisenko & Hargittai, 1996 ▸), the rotation is not hindered, and the mol­ecule can readily adopt different conformations.

**Figure 2:**
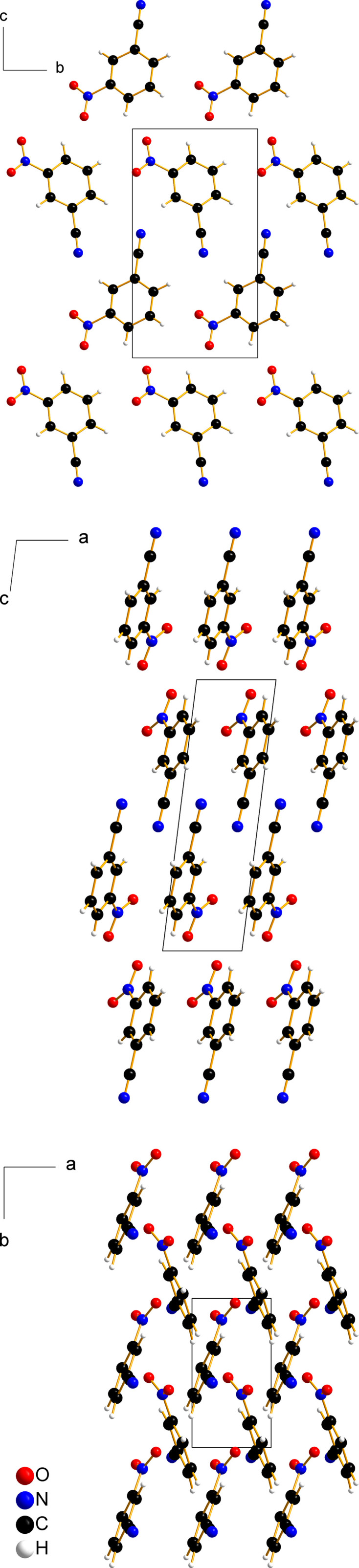
Packing diagrams and the unit cell of the title compound viewed along [100] (top), [010] (middle) and [001] (bottom).

The C—C and C—H bonds of the benzene ring span the ranges 1.3851 (19)–1.397 (2) Å and 0.91 (3)–0.96 (2) Å, respectively. The substituents are bonded to the benzene ring by a C—N bond of 1.4697 (19) Å in the case of the nitro group and a C—C bond of 1.447 (2) Å in the case of the nitrile group. The observed N—O distances of the nitro group are essentially equal [1.2258 (17) and 1.2262 (18) Å]. The length of the C≡N triple bond in the nitrile group is 1.141 (2) Å. In the crystal (Fig. 2 ▸), the mol­ecules are π-stacked along the shortest crystallographic axis, *a*, with an inter­planar distance of 3.3700 (9) Å.

Of the three positional isomers of nitro­benzo­nitrile, only the crystal structure of 4-nitro­benzo­nitrile has been previously reported (Cambridge Structural Database refcode PNBZNT; Higashi & Osaki, 1977 ▸). It also crystallizes in the Sohncke space group *P*2_1_ and the tilt angle of the nitro group out of the benzene ring plane (10.3°) is similar to the angle reported herein for the *meta* isomer [11.22 (6)°].

## Synthesis and crystallization

The title compound was obtained by decomposition of the corresponding diazo­nium salt in ethanol. The diazo­nium salt was synthesized by the previously published procedure (Mihelač *et al.*, 2021 ▸). *p*-Toluene­sulfonic acid monohydrate (570.7 mg; 3 mmol) was dissolved in 15 mL of ethyl acetate and 2-amino-5-nitro­benzo­nitrile (489.3 mg; 3 mmol) was added to the solution. The dropwise addition of *tert*-butyl nitrite (1068 µL, 9 mmol) resulted in the formation of a yellow sol­ution, which was stirred for 5 minutes at room temperature. The yellow precipitate of 2-cyano-4-nitro­benzene­diazo­nium tosyl­ate was obtained by filtration and washed thoroughly with ethyl acetate. This solid was then dissolved in 10 mL of EtOH and stirred for 3 days at room temperature. 3-Nitro­benzo­nitrile was isolated by filtration as an off-white solid. Single crystals were grown from a concentrated ethanol solution at −20 °C. A crystal suitable for single-crystal X-ray diffraction analysis was selected under a polarizing microscope and mounted on a MiTeGen Dual Thickness MicroLoop LD using Baysilone-Paste (Bayer-Silicone, mittelviskos).

## Refinement

Crystal data, data collection, and structure refinement details are summarized in Table 1 ▸. The positions of the hydrogen atoms were freely refined, including their isotropic displacement parameter *U* (Cooper *et al.*, 2010 ▸). The absolute structure was established based on the anomalous dispersion effects [Flack *x* = 0.02 (5); Hooft *y* = 0.05 (3); Parsons *et al.* (2013 ▸); Hooft *et al.* (2008 ▸)].

## Supplementary Material

Crystal structure: contains datablock(s) I. DOI: 10.1107/S2414314623008143/hb4451sup1.cif

Structure factors: contains datablock(s) I. DOI: 10.1107/S2414314623008143/hb4451Isup2.hkl

Supporting information file. DOI: 10.1107/S2414314623008143/hb4451Isup3.cml

CCDC reference: 2295676

Additional supporting information:  crystallographic information; 3D view; checkCIF report

## Figures and Tables

**Table 1:** Experimental details

Crystal data	
Chemical formula	C_7_H_4_N_2_O_2_
*M*_r_	148.12
Crystal system, space group	Monoclinic, *P*2_1_
Temperature (K)	100
*a*, *b*, *c* (Å)	3.73339 (4), 6.97307 (5), 12.87327 (9)
β (°)	97.1579 (8)
*V* (Å^3^)	332.52 (1)
*Z*	2
Radiation type	Cu *K*α
μ (mm^−1^)	0.95
Crystal size (mm)	0.28 × 0.06 × 0.04

Data collection	
Diffractometer	XtaLAB Synergy-S, Dualflex, Eiger2 R CdTe 1M
Absorption correction	Gaussian (*CrysAlis PRO*; Rigaku OD, 2023 ▸)
*T*_min_, *T*_max_	0.565, 1.000
No. of measured, independent and observed [*I* > 2σ(*I*)] reflections	10190, 1360, 1353
*R*_int_	0.025
(sin θ/λ)_max_ (Å^−1^)	0.629

Refinement	
*R*[*F* ^2^ > 2σ(*F* ^2^)], *wR*(*F* ^2^), *S*	0.024, 0.069, 1.09
No. of reflections	1360
No. of parameters	116
No. of restraints	1
H-atom treatment	All H-atom parameters refined
Δρ_max_, Δρ_min_ (e Å^−3^)	0.17, −0.17
Absolute structure	Flack *x* determined using 611 quotients [(*I* ^+^)−(*I* ^−^)]/[(*I* ^+^)+(*I* ^−^)] (Parsons *et al.*, 2013 ▸)
Absolute structure parameter	0.02 (5)

Computer programs: *CrysAlis PRO* (Rigaku OD, 2023 ▸), *OLEX2.solve* (Dolomanov *et al.*, 2009 ▸), *SHELXL2019/3* (Sheldrick, 2015 ▸), *OLEX2* (Dolomanov *et al.*, 2009 ▸) and *publCIF* (Westrip, 2010 ▸).

## full crystallographic data

### Crystal data

**Table:**

C_7_H_4_N_2_O_2_	*F*(000) = 152
*M**_r_* = 148.12	*D*_x_ = 1.479 Mg m^−^^3^
Monoclinic, *P*2_1_	Cu *K*α radiation, λ = 1.54184 Å
*a* = 3.73339 (4) Å	Cell parameters from 9014 reflections
*b* = 6.97307 (5) Å	θ = 3.5–75.7°
*c* = 12.87327 (9) Å	µ = 0.95 mm^−^^1^
β = 97.1579 (8)°	*T* = 100 K
*V* = 332.52 (1) Å^3^	Needle, colourless
*Z* = 2	0.28 × 0.06 × 0.04 mm

### Data collection

**Table:**

XtaLAB Synergy-S, Dualflex, Eiger2 R CdTe 1M diffractometer	1360 independent reflections
Radiation source: micro-focus sealed X-ray tube, PhotonJet (Cu) X-ray Source	1353 reflections with *I* > 2σ(*I*)
Mirror monochromator	*R*_int_ = 0.025
Detector resolution: 13.3333 pixels mm^-1^	θ_max_ = 75.9°, θ_min_ = 3.5°
ω scans	*h* = −4→4
Absorption correction: gaussian (CrysAlisPro; Rigaku OD, 2023)	*k* = −8→8
*T*_min_ = 0.565, *T*_max_ = 1.000	*l* = −16→16
10190 measured reflections	

### Refinement

**Table:**

Refinement on *F*^2^	Hydrogen site location: difference Fourier map
Least-squares matrix: full	All H-atom parameters refined
*R*[*F*^2^ > 2σ(*F*^2^)] = 0.024	*w* = 1/[σ^2^(*F*_o_^2^) + (0.0472*P*)^2^ + 0.0377*P*] where *P* = (*F*_o_^2^ + 2*F*_c_^2^)/3
*wR*(*F*^2^) = 0.069	(Δ/σ)_max_ < 0.001
*S* = 1.09	Δρ_max_ = 0.17 e Å^−^^3^
1360 reflections	Δρ_min_ = −0.17 e Å^−^^3^
116 parameters	Absolute structure: Flack *x* determined using 611 quotients [(*I*^+^)-(*I*^-^)]/[(*I*^+^)+(*I*^-^)] (Parsons *et al.*, 2013)
1 restraint	Absolute structure parameter: 0.02 (5)
Primary atom site location: iterative	

### Special details

**Table:**

Geometry. All esds (except the esd in the dihedral angle between two l.s. planes) are estimated using the full covariance matrix. The cell esds are taken into account individually in the estimation of esds in distances, angles and torsion angles; correlations between esds in cell parameters are only used when they are defined by crystal symmetry. An approximate (isotropic) treatment of cell esds is used for estimating esds involving l.s. planes.

### Fractional atomic coordinates and isotropic or equivalent isotropic displacement parameters (Å^2^)

**Table:**

	*x*	*y*	*z*	*U*_iso_*/*U*_eq_	
O1	0.2952 (4)	0.11413 (19)	0.94632 (9)	0.0316 (3)	
O2	0.5449 (3)	0.03136 (17)	0.81028 (9)	0.0294 (3)	
N1	0.2525 (4)	0.5665 (2)	0.45848 (11)	0.0323 (4)	
N2	0.3724 (3)	0.14375 (19)	0.85792 (10)	0.0199 (3)	
C1	0.1823 (4)	0.5195 (2)	0.65436 (11)	0.0192 (3)	
C2	0.2895 (4)	0.3452 (2)	0.70086 (12)	0.0179 (3)	
C3	0.2551 (4)	0.3250 (2)	0.80620 (11)	0.0171 (3)	
C4	0.1192 (4)	0.4678 (2)	0.86529 (12)	0.0195 (3)	
C5	0.0164 (4)	0.6403 (2)	0.81697 (12)	0.0215 (3)	
C6	0.0461 (4)	0.6674 (2)	0.71128 (13)	0.0218 (3)	
C7	0.2185 (4)	0.5471 (3)	0.54475 (12)	0.0236 (3)	
H2	0.387 (6)	0.249 (4)	0.6661 (17)	0.031 (6)*	
H4	0.108 (5)	0.445 (3)	0.9361 (16)	0.018 (5)*	
H6	−0.009 (5)	0.790 (4)	0.6782 (15)	0.024 (5)*	
H5	−0.071 (6)	0.738 (4)	0.8528 (17)	0.028 (5)*	

### Atomic displacement parameters (Å^2^)

**Table:**

	*U*^11^	*U*^22^	*U*^33^	*U*^12^	*U*^13^	*U*^23^
O1	0.0389 (7)	0.0334 (7)	0.0230 (5)	0.0024 (5)	0.0053 (5)	0.0092 (5)
O2	0.0321 (6)	0.0201 (6)	0.0368 (6)	0.0076 (5)	0.0073 (5)	0.0018 (5)
N1	0.0338 (8)	0.0387 (9)	0.0252 (6)	−0.0019 (6)	0.0064 (6)	0.0066 (6)
N2	0.0181 (6)	0.0192 (6)	0.0220 (6)	−0.0006 (5)	0.0014 (4)	0.0019 (5)
C1	0.0167 (6)	0.0217 (8)	0.0192 (6)	−0.0029 (5)	0.0016 (5)	0.0008 (6)
C2	0.0151 (7)	0.0184 (7)	0.0206 (6)	−0.0003 (5)	0.0034 (5)	−0.0018 (6)
C3	0.0148 (6)	0.0165 (7)	0.0199 (6)	−0.0017 (5)	0.0018 (5)	0.0006 (5)
C4	0.0165 (7)	0.0231 (7)	0.0190 (7)	−0.0010 (6)	0.0021 (5)	−0.0023 (6)
C5	0.0183 (7)	0.0202 (7)	0.0258 (7)	0.0016 (6)	0.0020 (5)	−0.0070 (6)
C6	0.0181 (7)	0.0190 (8)	0.0275 (7)	0.0005 (6)	−0.0004 (5)	0.0013 (6)
C7	0.0214 (7)	0.0246 (7)	0.0247 (7)	−0.0022 (6)	0.0027 (6)	0.0029 (6)

### Geometric parameters (Å, º)

**Table:**

O1—N2	1.2258 (17)	C2—H2	0.91 (3)
O2—N2	1.2262 (18)	C3—C4	1.387 (2)
N1—C7	1.141 (2)	C4—C5	1.386 (2)
N2—C3	1.4697 (19)	C4—H4	0.93 (2)
C1—C2	1.392 (2)	C5—C6	1.392 (2)
C1—C6	1.397 (2)	C5—H5	0.91 (2)
C1—C7	1.447 (2)	C6—H6	0.96 (2)
C2—C3	1.3851 (19)		
			
O1—N2—O2	123.75 (13)	C4—C3—N2	118.53 (12)
O1—N2—C3	118.31 (12)	C3—C4—H4	118.7 (12)
O2—N2—C3	117.93 (12)	C5—C4—C3	118.46 (13)
C2—C1—C6	121.55 (14)	C5—C4—H4	122.8 (12)
C2—C1—C7	118.61 (14)	C4—C5—C6	120.34 (14)
C6—C1—C7	119.84 (14)	C4—C5—H5	121.5 (14)
C1—C2—H2	123.1 (14)	C6—C5—H5	118.1 (14)
C3—C2—C1	116.93 (13)	C1—C6—H6	119.6 (12)
C3—C2—H2	119.9 (14)	C5—C6—C1	119.42 (14)
C2—C3—N2	118.16 (12)	C5—C6—H6	120.9 (12)
C2—C3—C4	123.30 (13)	N1—C7—C1	178.69 (17)
			
O1—N2—C3—C2	−169.59 (13)	C2—C1—C6—C5	0.0 (2)
O1—N2—C3—C4	11.0 (2)	C2—C3—C4—C5	−0.8 (2)
O2—N2—C3—C2	11.01 (19)	C3—C4—C5—C6	0.7 (2)
O2—N2—C3—C4	−168.36 (13)	C4—C5—C6—C1	−0.3 (2)
N2—C3—C4—C5	178.55 (12)	C6—C1—C2—C3	0.0 (2)
C1—C2—C3—N2	−178.92 (13)	C7—C1—C2—C3	179.18 (13)
C1—C2—C3—C4	0.4 (2)	C7—C1—C6—C5	−179.21 (13)
